# Sex Differences in Non-Acute Myocardial Infarction Cardiogenic Shock: Insights from the Northwell-Shock Registry

**DOI:** 10.3390/jcm14124274

**Published:** 2025-06-16

**Authors:** Shuojohn Li, Abduljabar Adi, Marcy Miller, Fouad Sakr, Jack Jnani, Emily A. Rodriguez, Samuel Tan, Moein Bayat Mokhtari, Ramsis Ramsis, Rebecca Smoller, Gerin R. Stevens, Jaime Hernandez-Montfort, Matthew Griffin, Matthew Pierce, Miguel Alvarez Villela

**Affiliations:** 1 Department of Medicine, North Shore University Hospital, Northwell Health, Manhasset, NY 11030, USA; sli13@northwell.edu (S.L.); jjnani@northwell.edu (J.J.); 2 Cardiovascular Institute, Northwell Health, New Hyde Park, NY 11042, USA; aadi@northwell.edu (A.A.); mmiller41@northwell.edu (M.M.); rsmoller1@northwell.edu (R.S.); gstevens1@northwell.edu (G.R.S.); mgriffin9@northwell.edu (M.G.); mpierce1@northwell.edu (M.P.); 3 Department of Medicine, Staten Island University Hospital, Northwell Health, Staten Island, NY 10305, USA; fsakr@northwell.edu; 4 Department of Medicine, Lenox Hill Hospital, Northwell Health, New York, NY 10075, USA; erodriguez45@northwell.edu (E.A.R.); mbayatmokhta@northwell.edu (M.B.M.); rramsis@northwell.edu (R.R.); 5 Department of Medicine, Mount Sinai Morningside/West, New York, NY 10025, USA; stan2@northwell.edu; 6 Advanced Heart Disease, Recovery and Replacement Program, Baylor Scott & White Health, Temple, TX 76508, USA; jaime.hernandezmontfort@bswhealth.org

**Keywords:** cardiogenic shock, gender differences, mechanical circulatory support, pulmonary artery catheter, shock etiology

## Abstract

**Background:** While the role of sex-based differences in acute myocardial infarction-related cardiogenic shock (AMI-CS) is well described, their relevance among patients with non-acute myocardial infarction-related CS (nonAMI-CS) is less well known. **Methods:** Adult patients treated for cardiogenic shock (CS) between 2016 and 2022 across eleven hospitals within our health system were included. NonAMI-CS etiologies were classified as heart failure or secondary CS (valvular and arrhythmia-triggered). Stratification by sex was used to compare characteristics, management strategies, and outcomes between men and women. Logistic regression models were used to examine the effect of clinical characteristics and management strategies on hospital mortality by sex, along with the effect of sex on different management strategies. **Results:** Of 2256 patients, women comprised the minority (36%) and also exhibited older age and more comorbidities. Both sexes displayed similar presenting shock stages, according to those established by the Society for Cardiovascular Angiography and Interventions (SCAI). Valvular shock was the only etiology which was more prevalent in women. Women received fewer invasive interventions, including treatment with pulmonary artery catheters (44% vs. 53%; *p* < 0.01) and mechanical circulatory support devices (15% vs. 22%; *p* < 0.01). While sex was not independently associated with increased mortality (OR = 1.16, 95% CI = 0.68–1.96), women were more likely to be discharged to skilled nursing facilities (SNF). **Conclusions:** Although women with nonAMI-CS exhibit a higher risk profile and undergo fewer invasive procedures, their survival rate is comparable to that of men. However, women are more likely to require SNF care upon discharge.

## 1. Introduction

Cardiogenic shock (CS) is a complex syndrome defined by severe impairment of cardiac function leading to reduced cardiac output, tissue hypoperfusion, and multi-organ dysfunction [1]. Hospital mortality remains as high as 30–50% for CS patients despite more aggressive care, including higher utilization of mechanical circulatory support (MCS) [2,3]. The diversity in etiologies and the variation in disease severity at the time of presentation have hampered the development of uniformly effective therapies [4].

There is a growing understanding that patient sex plays an important role in defining clinical presentation and outcomes, especially among patients with AMI-related CS (AMI-CS) [5,6]. Whether sex also plays a role among patients with non-AMI-related CS (nonAMI-CS) is less well described.

This knowledge gap is particularly important because nonAMI-CS represents a more heterogeneous patient population in which the variations in presentation and management strategies are particularly wide. It also accounts for a majority of cases in contemporary CS registries [7,8]. Understanding the sex-related differences in this group can help to better describe the epidemiology of CS, facilitating the development of sex-specific management strategies and improving clinical trial design.

## 2. Materials and Methods

### 2.1. Study Population

The registry is composed of data from 11 hospitals within the Northwell Health system in the New York metropolitan area, including four cardiac surgery sites within our 23-hospital network. All patients over 18 years of age discharged from these sites between January 2016 and August 2022 with a principal or secondary diagnosis of CS are included. CS was identified using the International Classification of Disease (ICD)-10 code R57.0 [9].

Data were collected via an automated process through electronic health records, obtaining information directly from the chart, including demographics, comorbidities, laboratory results, level of care at discharge, treatments provided, and hospital outcomes using ICD-10 procedural and diagnostic codes (Supplemental Tables S1 and S2). The billing of ICD-10 codes follows a uniform and centralized process within our hospital system, ensuring diagnostic code consistency. Comorbidities including diabetes mellitus, hypertension, chronic kidney disease (CKD), heart failure (HF), coronary artery disease (CAD), and atrial fibrillation were defined by the presence of the corresponding ICD-10 diagnosis code [10] in patients’ electronic medical records (Supplemental Table S1). Baseline vital signs and laboratory values were defined as the first available laboratory value results within 24 h of hospital presentation. When analyzing adverse outcomes, advanced MCS was defined as the use of venoarterial extracorporeal membrane oxygenation (VA-ECMO) (Maquet Cardiopulmonary AG, Hirrlingen, Germany, or CentriMag, Abbott Laboratories, Abbott Park, IL, USA) and/or microaxial flow pump (mAFP) (Impella devices, Abiomed, Danvers, MA, USA) devices. The vasoactive-inotropic score (VIS) was calculated based on the number and dosage of inotropes and vasopressors at admission (VIS 0 h) and at the time of maximal dosing (VIS max) [11]. The significant complications included major bleeding, defined using the Bleeding Academic Research Consortium (BARC) criteria; arterial thrombosis requiring surgical intervention; new renal replacement therapy (RRT); and sepsis, defined as clinical worsening, with a documented or suspected infection. Information regarding these complications was extracted manually from the charts.

### 2.2. Shock Etiologies and Severity of Shock

The primary etiology of CS was assigned based on ICD-10 codes for principal discharge diagnosis following the classification proposed by the Shock Academic Research Consortium (SHARC) [12]: (1) AMI-CS; (2) nonAMI-CS, including de novo and acute on chronic heart failure (ACHF), as well as “secondary CS” due to arrhythmias and valvular disease; (3) other etiologies, including post-cardiotomy CS, mixed septic-CS, pulmonary embolism, as well as other non-cardiac causes which have been previously described [9]. For this analysis, only patients within the nonAMI-CS category were included (Supplemental Figure S1). Specific sub-categories were described within the valvular and arrhythmic etiologies. A complete list of the ICD-10 codes assigned to each etiologic group is provided in the Supplemental Material (Supplemental Tables S1 and S2).

The severity of CS is described according to the Society for Cardiovascular Angiography and Interventions (SCAI) stages classification using the objective criteria previously described by the cardiogenic shock working group (CSWG) [13,14]. The APACHE IV score at the time of intensive care unit (ICU) admission was calculated for patients admitted to an intensive care unit (ICU) at any point during their hospital stay [15].

### 2.3. Statistical Analysis

Data analysis was conducted using RStudio version 4.4.1, while graphs were generated using GraphPad Prism version 10.3.1. Missing values were handled through multiple imputation via chained equation (using the “mice” R package). Continuous variables were reported as the mean ± SD for normally distributed data or as the median (25th percentile, 75th percentile) for non-normally distributed data. Categorical variables were summarized as frequencies and percentages. *p* values of <0.05 were chosen for statistical significance and adjusted by Bonferroni correction, when needed.

Comparison of continuous variables was performed using t-tests or Wilcoxon rank-sum tests. Categorical variables were compared using Chi-square tests. Multivariate logistic regression models were constructed to examine the effects of sex on in-hospital mortality and management strategies, as well as the sex-specific predictors of mortality.

The primary outcome was in-hospital mortality. Secondary outcomes included in-hospital mortality stratified by the specific etiology of CS, disposition at hospital discharge for survivors, and rate of major complications in patients receiving advanced MCS (e.g., mAFP and/or VA-ECMO).

## 3. Results

### 3.1. Baseline Characteristics

A total of 2256 patients were included in this analysis (Supplemental Figure S1), with women comprising 36% of the cohort. The women were older, exhibited a smaller body surface area, and displayed more comorbidities based on the Charlson Comorbidity Index than men [10]. Women had a lower prevalence of previous CAD and CKD but a lower glomerular filtration rate (eGFR) on presentation. Previous history of HF was highly prevalent in both sexes (74% of women vs. 77% of men; *p* = 0.10) (Table 1).

According to transthoracic echocardiogram, women exhibited higher left ventricular (LV) ejection fractions and smaller LV end-diastolic diameters. Notable differences in laboratory analyses included lower alanine transferase, total bilirubin, and hemoglobin levels, as well as a higher platelet count, in women. Lactate levels at presentation were similar in both sexes (Table 1).

### 3.2. Specific Etiologies and Shock Severity

**Figure 1:**
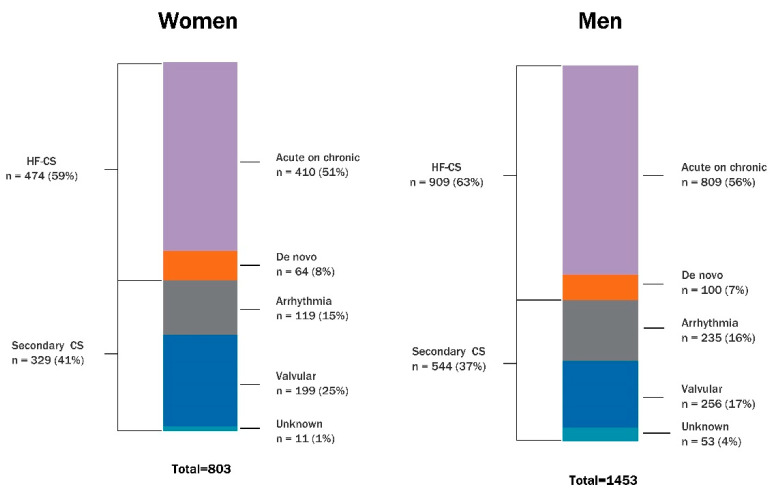
Etiologies of non-acute myocardial infarction-related cardiogenic shock by patient sex. *CS = cardiogenic shock; HF-CS = heart failure-related cardiogenic shock*.

Women were more frequently affected by valvular CS (VCS) (23% vs. 15%; adjusted *p* < 0.01), while heart failure-associated-CS (HF-CS) and arrhythmia-triggered CS were similarly prevalent between women and men. Subgroup analysis was also performed to compare the prevalence of specific sub-etiologies. Within HF-CS, the prevalence of ACHF and de novo HF was comparable between sexes. For arrhythmia-triggered CS, women were more affected than men by bradyarrhythmia (42% vs. 20% of total arrhythmia-triggered CS; adjusted *p* < 0.01) but less by ventricular arrhythmia (24% vs. 51%; adjusted *p* < 0.01) (Table 2 and Figure 1). Among VCS patients, women were more frequently affected by mitral regurgitation (37% vs. 21% of total VCS; adjusted *p* < 0.01) but less by aortic regurgitation (5% vs. 15%; adjusted *p* < 0.01) than were men, while the prevalence of aortic stenosis, mitral stenosis, right-sided valve disease, prosthetic valve diseases, and infective endocarditis were comparable between sexes (Table 2 and Supplemental Figure S2).

CS severity was measured using the SCAI-CSWG stages [13,14] and the APACHE score at ICU admission [15]. No significant sex-based differences were seen in the initial SCAI stage distribution or average APACHE scores. All patients progressed to at least SCAI-CSWG stage B shock during their stays (61% stage E, 22% stage D, 11% stage C, 4% stage B, and 2% not recorded), and there was no sex difference in regards to maximal SCAI-CSWG stages reached during the CS hospitalization (Table 1 and Supplemental Figure S3).

### 3.3. Management Strategies

**Figure 2:**
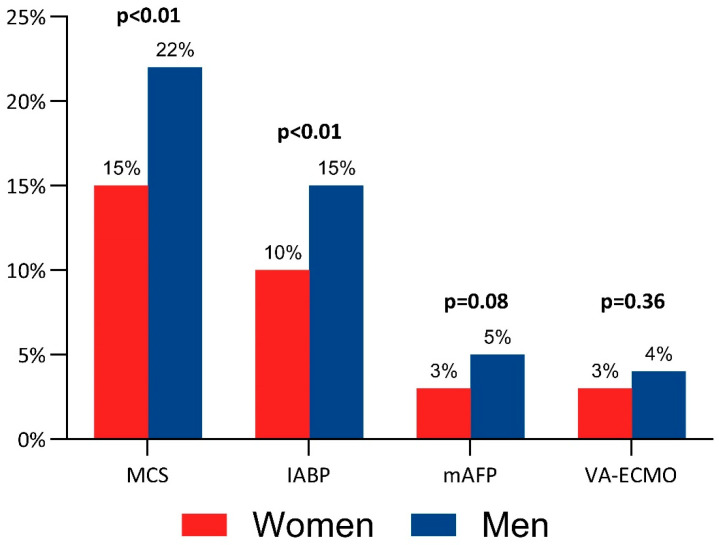
Sex difference in the use of mechanical circulatory support in non-acute myocardial infarction-related cardiogenic shock. *IABP = intra-aortic balloon pump, mAFP = microaxial flow pump, MCS = mechanical circulatory support, and VA-ECMO = venoarterial extracorporeal membrane oxygenation*.

Women had lower rates of pulmonary artery catheter (PAC) use for invasive hemodynamic monitoring (44% vs. 53%; *p* < 0.01) and the use of MCS devices (15% vs. 22%; *p* < 0.01), driven mostly by the less frequent use of the intra-aortic balloon pump (IABP). mAFP and VA-ECMO were used with equal frequency in both sexes (Table 3 and Figure 2). Among patients who received Impella treatment (Abiomed, Danvers, MA), the device types used comprised Impella CP in 76%, 5.0 in 7%, 2.5 in 4%, 5.5 in 3%, RP in 3%, and the type was unrecorded in 7% of patients.

Regarding other ICU therapies, women received fewer RRTs, more blood transfusions, and similar rates of mechanical ventilation, while no differences were observed in the overall use of vasopressors or inotropes, nor in regards to initial or maximal VIS. There was a slightly higher use of milrinone in men and of dopamine in women. Coronary artery bypass graft surgery (CABG) occurred less often in women compared to men, while percutaneous and surgical valvular interventions were more frequent among women. Other invasive management procedures such as left heart catheterization (LHC) and percutaneous coronary intervention (PCI) were used equally in both sexes. The overall use of heart replacement therapies was low, but the frequency of durable left ventricular assist device (LVAD) implantation was lower in women than in men, while heart transplantation was used at similar rates in both sexes (Table 3).

In multivariate analysis adjusting for age, body surface area (BSA), lactate levels, eGFR, and history of hypertension and diabetes, patients receiving blood transfusion were more likely to be women (OR = 1.63, 95% CI: 1.29–2.07), while IABP (OR = 0.65, 95% CI: 0.47–0.89), PAC (OR = 0.77, 95% CI: 0.62–0.95), CABG (OR = 0.43, 95% CI: 0.26–0.68) and durable LVAD recipients (OR = 0.31, 95% CI: 0.15–0.63) were all less likely to be women (Supplemental Figure S4).

### 3.4. Complications in Advanced Mechanical Circulatory Support

The frequency of advanced MCS device use, defined as mAFP, VA-ECMO or the combination of the two, was low overall, with no sex-based difference. In addition, no differences were found in the rates of major bleeding (BARC 3–5), arterial thrombosis requiring surgery, the need for RRT, or sepsis in this subgroup (Table 4).

### 3.5. Hospital Outcomes

Women and men displayed similar overall in-hospital mortality. On univariate analysis, women experienced a high mortality rate in HF-CS (33% vs. 25%; adjusted *p* = 0.02) but a similar mortality rate to that of men in VCS and arrhythmia-triggered CS. There was no sex difference in regards to length of hospital stay. However, women were less likely to be discharged to home compared to men and more likely to be discharged to rehabilitation or skilled nursing facilities (SNF), as well as hospice facilities (Table 5). 

Female sex is not an independent predictor of mortality in multivariate analysis (OR = 1.16, 95% CI = 0.68–1.96). However, multivariate logistic regression models constructed separately for women and men revealed different mortality predictors between the sexes. Significant multivariable factors associated with higher mortality included a history of hypertension (OR = 1.65, 95% CI = 1.07–2.54) and HF-CS as the etiology of shock (OR = 1.54, 95% CI = 1.01–2.34) in women, while a higher BSA (OR = 1.16, 95% CI = 1.01–1.32) and treatment with vasopressors (OR = 1.46, 95% CI = 1.01–2.10) were associated with higher mortality in men.

Factors associated with lower mortality included durable LVAD implantation (OR = 0.23, 95% CI = 0.08–0.71) and treatment with PCI (OR = 0.35, 95% CI = 0.13–0.96) in men, and the use of PAC (OR = 0.55, 95% CI = 0.35–0.87) in women.

**Figure 3:**
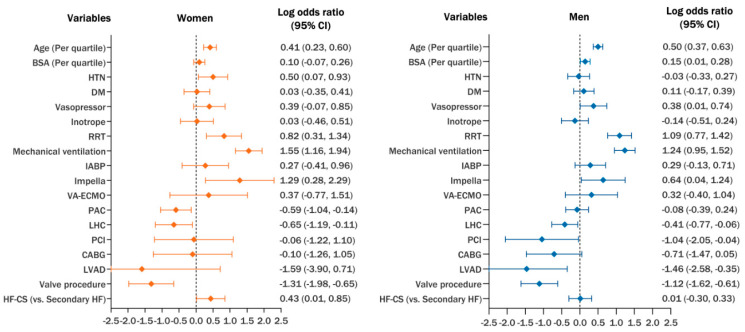
Multivariate logistic regression model of mortality-associated factors in women and men treated with non-acute myocardial infarction-related cardiogenic shock. *BSA = body surface area, CABG = coronary artery bypass graft, DM = diabetes mellitus, HF = heart failure, HF-CS = heart failure-related cardiogenic shock, HTN = hypertension, IABP = intra-aortic balloon pump, LHC = left heart catheterization, LVAD = left ventricular assist device, PAC = pulmonary artery catheter, PCI = percutaneous coronary intervention, RRT = renal replacement therapy, and VA-ECMO = venoarterial extracorporeal membrane oxygenation*.

Older age, the need for RRT or mechanical ventilation, as well as treatment with mAFP, were all associated with increased mortality in both sexes (Figure 3). These factors remained as significant predictors of mortality in a multivariate adjusted model excluding CS etiologies as covariates (Supplemental Figure S5).

## 4. Discussion

**Figure 4:**
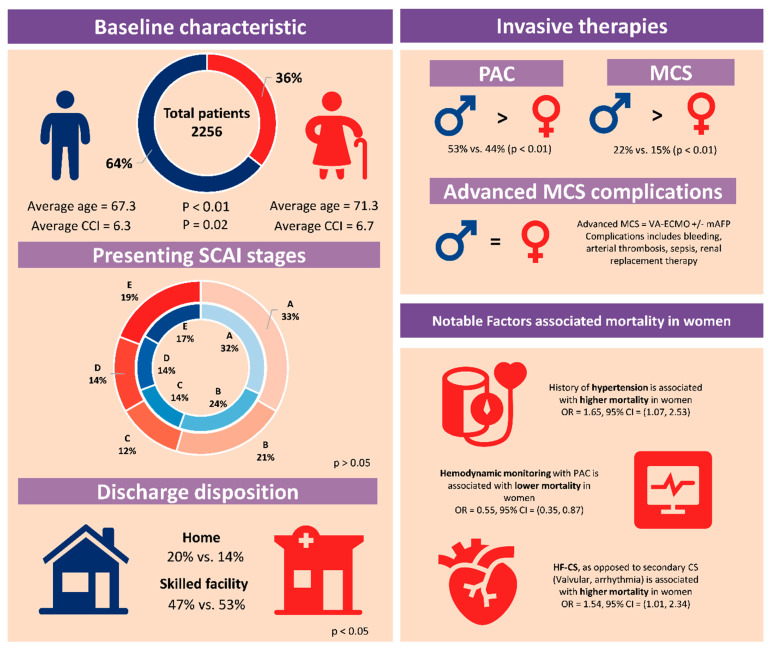
Central illustration. CCI = Charlson comorbidities index, PAC = pulmonary artery catheter, MCS = mechanical circulatory support, VA-ECMO = venoarterial extracorporeal membrane oxygenation, mAFP = microaxial flow pump, and HF-CS = heart failure-related cardiogenic shock.

This study aimed to describe the sex-related differences in nonAMI-CS across a large and diverse cohort of patients treated within a multi-tier healthcare system in the New York metropolitan area. Our main findings are summarized in the central illustration (Figure 4). The principal novelty of our study is the detailed description of specific etiologies constituting the nonAMI-CS etiology, according to the classification proposed by the SHARC [7]. Our findings further the understanding of CS epidemiology and help refine the definition of CS among patients without AMI, providing insight into the sex-related factors influencing management and outcomes in this population.

HF-CS, considered the only primary etiology of nonAMI-CS, was the most common etiology in our cohort, and the prevalence of de novo vs. ACHF as a trigger was comparable between both sexes. This differs from what had been reported by other registries, which suggested that de novo HF is more prevalent in women. However, those registries did not subclassify the etiologies of nonAMI-CS, and it is possible that certain etiologies like VCS or arrhythmia-triggered CS were grouped within HF-CS, altering the relative prevalence of each subgroup [7,8,16,17]. Among the secondary etiologies, VCS was the most common and was more prevalent among women. Moreover, women were more frequently affected by mitral regurgitation and less by aortic regurgitation, reflecting the overall sex-based distribution pattern of valvular heart disease seen in the general population [18]. In this study, we observed a much lower mortality among patients with VCS compared to that noted in a recent study from a single-center cardiac intensive care unit population (13–14% vs. 20–28%) [19]. In fact, patients of both sexes exhibiting VCS had the lowest mortality among all the etiologies in our study. Variations in the included population and in the definition of VCS may explain these differences. Notably, a large proportion of patients in this study underwent percutaneous valvular interventions (23% of all valvular interventions in women, 16% of all valvular interventions in men), suggesting that transcatheter valvular therapies play an increasing role in CS. This has also been reported in other recent studies [19].

Arrhythmia-triggered CS was also similarly prevalent in both sexes, but with important differences in the types of arrhythmias triggering CS. These trends again align with established epidemiological patterns noted in regards to arrhythmia prevalence by sex [20]. A detailed breakdown of the type of arrhythmias triggering CS by sex is provided in the Supplementary Material (Supplemental Figure S2).

Notable sex-based differences in management strategies were also observed in this study. Women less frequently received PAC, MCS, CABG, and LVAD. These management differences may be driven by higher perceived procedural risk among women, given their older age and higher comorbidity burden, as well as potential implicit bias among clinicians, which was not captured in our study. Similar trends of sex-based differences in invasive therapies utilization have also been reported by the CSWG and the Critical Care Cardiology Trials Network (CCCTN) [7,8]. However, in contrast with those studies, women in our cohort received comparable rates of advanced MCS, namely mAFP and VA-ECMO, with lower rates of IABP. Also in contrast to the results of those studies, the complication rates seen among women with advanced MCS in this study were similar to those seen in men. Variations in adverse event definition, as well as practice patterns unique to our health system, might explain some of these differences between registries.

The differences seen in this study between sexes with regards to PAC are worth mentioning. While the role of PAC in CS management remains controversial and its use was lower in women than in men in this and other prior reports [7,8], its use was associated with lower adjusted mortality among women. This effect may be restricted to the nonAMI-CS etiology, since the opposite trend, favoring PAC use in men to improve survival, was seen when considering all etiologies together in the CSWG registry [7]. A specific focus on sex differences in regards to the effects of PAC use is needed in future research to reduce disparities in care.

While mortality was higher for women in the HF-CS group, the rates of all other specific etiologies were similar for both sexes. The overall unadjusted hospital mortality was comparable between men and women when considering all etiologies, and multivariable analysis revealed that sex was not an independent predictor of mortality, despite baseline differences.

However, specific factors associated with mortality did differ by sex. Notably, while larger body surface area led to higher mortality risk in men, it had no effect among women. Meanwhile, treatment with mAFP devices, mostly the Impella-CP (Abiomed, Danvers, MA, USA), was associated with increased mortality in both sexes. The effect of the mAFP treatment on patient survival has been variable in nonAMI-CS. Although the recent DanGer Shock trial showed a survival benefit, this study included only patients with STEMI-CS [21]. Our findings underscore the need for further research evaluating device-specific mortality in nonAMI-CS, particularly focusing on newer device types such as the Impella 5.5 (Abiomed, Danvers, MA, USA), which is increasingly used in the HF-CS population [22,23].

Finally, women were less likely to return home and more often required discharge to SNF or hospice. This difference likely reflects the older age and higher comorbidity burden observed in women. The impact of SNF discharge on long-term mortality and morbidity among CS survivors remains poorly understood. Prior studies have suggested that SNF discharge may be associated with reduced 30-day readmission rates in patients with nonAMI-CS, but increased long-term mortality in those with AMI-CS. Further research is needed to evaluate the impact of discharge disposition on outcomes among CS survivors [24,25].

## 5. Conclusions

NonAMI-CS is a heterogenous etiologic group with important sex-related differences, including in regards to the specific triggers of CS, management strategy selection by clinicians, and clinical factors associated with adverse outcomes. Women in the study exhibited older age and a higher comorbidity burden but presented with a similar severity of CS, based on SCAI shock stages. Compared to men, women received less invasive therapies, including PAC for hemodynamic monitoring, which nevertheless was associated with improved survival in women after adjusted analysis, denoting the existing disparities in care and their potential effects on outcomes. Despite these differences, both sexes displayed an overall similar survival, but women exhibited a higher need for SNF after discharge.

## 6. Limitations

This study relies on the accuracy of administrative data to identify patients with CS. However, multiple contemporary CS registries have used a similar approach, and prior studies examining the accuracy of the R57.0 ICD-10 code to identify CS have reported its positive predictive value to be as high as 93% [26].

We also rely on administrative data to classify the different CS etiologies and collect their relevant management procedures and outcomes. The accuracy of these codes is less well-studied. Additionally, complications were not assessed for patients treated with IABP or medical therapy alone. However, prior studies have shown a low incidence of major complications in these patients with CS [27]. Finally, our study did not collect data regarding readmissions or outcomes beyond initial discharge, and hence, we cannot assess the mid- and long-term course of these patients.

## Figures and Tables

**Table 1:** Baseline demographics, comorbidities, admission characteristics, and laboratory data.

Parameter	Women *n* = 803	Men *n* = 1453	*p* Value
**Age (year)**	71.3 ± 14.6	67.7 ± 13.8	<0.01
**BMI (kg/m^2^)**	30.1 ± 10.1	29.6 ± 9.7	0.22
**BSA (m^2^)**	1.84 ± 0.33	2.06 ± 0.34	<0.01
**Comorbidities (%)**			
** Diabetes mellitus**	322 (40)	624 (43)	0.19
** Hypertension**	551 (69)	976 (67)	0.48
** Chronic kidney disease**	322 (40)	673 (46)	<0.01
** Heart failure**	595 (74)	1122 (77)	0.10
** Coronary artery disease**	264 (33)	681 (47)	<0.01
** Atrial fibrillation**	403 (50)	742 (51)	0.69
**CCI**	6.7 ± 3.66	6.3 ± 3.66	0.02
**Cardiac arrest at admission**	18 (2)	45 (3)	0.24
**Admission APACHE score**	76.1 ± 32.4	74.2 ± 32.1	0.19
**Admission SCAI stage (%)**			0.40
** A**	256 (32)	442 (30)	
** B**	162 (20)	338 (23)	
** C**	94 (12)	190 (13)	
** D**	111 (14)	194 (13)	
** E**	147 (18)	232 (16)	
**Not recorded**	33 (4)	57 (4)	
**EF (%) (*n* = 346 vs. 657)**	30 (20, 55)	23 (15, 35)	<0.01
**LVEDD (cm) (*n* = 348 vs. 705)**	4.8 (4.2, 5.6)	5.8 (5.0, 6.4)	<0.01
**Laboratory**			
** Lactate (mmol/L)**	2.2 (1.7, 3.8)	2.2 (1.7, 3.8)	0.71
** Creatinine (mg/dL)**	1.55 (1.02, 2.53)	1.82 (1.28, 2.87)	<0.01
** eGFR (mL/min/1.73 m^2^)**	41.2 (23.5, 69.0)	45.2 (27.6, 69.6)	<0.01
** ALT (IU/L)**	28 (16, 66)	33 (19, 87)	<0.01
** Bilirubin (mg/dL)**	0.8 (0.5, 1.4)	1.0 (0.6, 1.8)	<0.01
** Sodium (mEq/L)**	137 (133, 140)	137 (133, 139)	0.24
** Hemoglobin (g/dL)**	11.1 (9.6, 12.5)	12.1 (10.2, 13.6)	<0.01
** WBC (k/uL)**	9.3 (6.4, 13.3)	8.8 (6.6, 12.3)	0.16
** Platelet (k/uL)**	189 (133, 246)	177 (126, 234)	<0.01
** Albumin (g/dL)**	3.4 (3, 3.8)	3.4 (3.1, 3.8)	0.1

*ALT = alanine transaminase, BMI = body mass index, BSA = body surface area, CCI = Charlson Comorbidity Index, EF = ejection fraction, eGFR = estimated glomerular filtration rate, LVEDD = left ventricular end-diastolic diameter, SCAI = Society of Cardiovascular Angiography and Interventions, and WBC = white blood cell.*

**Table 2:** Etiologies of cardiogenic shock.

Etiology (%)	Women *n* = 803	Men *n* = 1453	Adjusted *p* Value
**Heart failure-related CS**	474 (59)	909 (63)	0.30
** Acute on chronic**	410 (86)	809 (89)	
** De novo**	64 (14)	100 (11)	
**Arrhythmia-triggered CS**	119 (15)	235 (16)	1.0
** Atrial**	38 (32)	63 (27)	1.0
** Ventricular**	29 (24)	121 (51)	<0.01
** Bradyarrhythmia**	50 (42)	47 (20)	<0.01
** Not recorded**	2 (2)	4 (2)	
**Valvular CS**	199 (25)	256 (17)	<0.01
** Aortic stenosis**	38 (19)	65 (25)	1.0
** Aortic regurgitation**	9 (5)	38 (15)	<0.01
** Mitral stenosis**	5 (2)	2 (1)	1.0
** Mitral regurgitation**	73 (37)	53 (21)	<0.01
** Prosthetic valve**	7 (4)	24 (9)	0.24
** Right-sided valve**	5 (2)	5 (2)	1.0
** Multivalvular**	42 (21)	31 (12)	0.10
** Infectious endocarditis**	18 (9)	37 (14)	0.87
** Not recorded**	2 (1)	1 (0.3)	
**Not recorded**	11 (1)	53 (4)	

*CS = Cardiogenic shock.*

**Table 3:** Non-acute myocardial infarction-related cardiogenic shock management.

Intervention (%)	Women *n* = 803	Men *n* = 1453	Adjusted *p* Value
**Catecholamine**	287 (36)	515 (35)	0.89
**Any inotrope**	322 (40)	608 (42)	0.42
** Dobutamine**	234 (29)	435 (30)	0.72
** Milrinone**	188 (23)	405 (28)	0.01
** Dopamine**	32 (4)	35 (2)	0.02
**VIS score**			
** At 0 h (*n* = 189 vs. 323)**	10 (5, 25)	8 (4, 24)	0.19
** At max (*n* = 366 vs. 623)**	21 (9, 65)	22 (8, 52)	0.44
**Any MCS**	117 (15)	322 (22)	<0.01
** IABP**	80 (10)	215 (15)	<0.01
** mAFP**	28 (3)	74 (5)	0.08
** VA-ECMO**	28 (3)	62 (4)	0.36
**LVAD**	12 (1)	54 (4)	<0.01
**Transplant**	14 (2)	22 (2)	0.68
**Pulmonary artery catheter**	353 (44)	773 (53)	<0.01
**Left heart catheterization**	191 (24)	385 (26)	0.16
**PCI**	23 (3)	49 (3)	0.59
**CABG**	29 (4)	93 (6)	<0.01
**Valvular procedure**	172 (21)	258 (18)	0.03
** Surgical**	117 (68)	155 (60)	
** Percutaneous**	39 (23)	42 (16)	
** Unrecorded**	16 (9)	61 (24)	
**Blood transfusion**	277 (34)	406 (28)	<0.01
**Mechanical ventilation**	272 (34)	499 (34)	0.82
**NIPPV**	180 (22)	355 (24)	0.28
**RRT**	107 (13)	240 (17)	0.04

*CABG = coronary artery bypass graft, IABP = intra-aortic balloon pump, LVAD = durable left ventricular assist device, mAFP = microaxial flow pump, MCS = mechanical circulatory support, NIPPV = noninvasive positive pressure ventilation, PCI = percutaneous coronary intervention, RRT = renal replacement therapy, VA-ECMO = venoarterial extracorporeal membrane oxygenation, and VIS = vasoactive-inotropic score.*

**Table 4:** Major complications in non-acute myocardial infarction-associated cardiogenic shock patients treated with advanced mechanical circulatory support.

Advanced MCS Complications (%)	Women *n* = 46	Men *n* = 117	*p* Value
**Impella only**	***n* = 18**	***n* = 55**	
BARC 3–5 bleeding	1 (6)	4 (7)	1.0
Arterial thrombosis	1 (6)	2 (4)	1.0
Transfusion	4 (22)	9 (16)	0.83
RRT	7 (56)	13 (24)	0.34
Sepsis	1 (6)	7 (13)	0.68
**VA-ECMO only**	***n* = 18**	***n* = 45**	
BARC 3–5 bleeding	0 (0)	4 (9)	0.46
Arterial thrombosis	0 (0)	0 (0)	NA
Transfusion	1 (6)	11 (24)	0.46
RRT	2 (11)	6 (13)	1.0
Sepsis	1 (6)	2 (4)	1.0
**Impella and VA-ECMO**	***n* = 10**	***n* = 17**	
BARC 3–5 bleeding	3 (30)	3 (18)	0.79
Arterial thrombosis	1 (10)	1 (6)	1.0
Transfusion	1 (10)	6 (35)	0.32
RRT	3 (30)	3 (18)	0.79
Sepsis	2 (20)	2 (12)	0.98

*BARC = Bleeding Academic Research Consortium, MCS = mechanical circulatory support, RRT = renal replacement therapy, and VA-ECMO = venoarterial extracorporeal membrane oxygenation.*

**Table 5:** Hospital outcome of cases of non-acute myocardial infarction-related cardiogenic shock.

Outcome	Women *n* = 803	Men *n* = 1453	Adjusted *p* Value
**Disposition at discharge (%)**			<0.01
** Home**	110 (14)	290 (20)	
** Rehab or skilled facility**	379 (47)	644 (44)	
** Hospice**	44 (6)	45 (3)	
** Unrecorded**	50 (6)	112 (8)	
**Length of stay (day)**	12 (6, 20)	12 (6, 21)	0.39
**In-hospital mortality (%)**	220 (27)	362 (25)	0.20
** HF-CS**	155 (33)	231 (25)	0.02
** Arrhythmia-triggered CS**	30 (25)	69 (29)	1.0
** VCS**	30 (14)	43 (13)	1.0
** Etiology not recorded**	5	19	

*CS = cardiogenic shock, HF-CS = heart failure related cardiogenic shock, and VCS = valvular cardiogenic shock.*

## Data Availability

The raw data supporting the conclusions of this article will be made available by the authors on request.

## Supplementary Materials

The following supporting information can be downloaded at: https://www.mdpi.com/article/10.3390/jcm14124274/s1, Table S1: ICD-10 Admission & Principal Diagnosis Codes Included in Each Etiology Group. ICD = international classification of disease; AMI = acute myocardial infarction; AHF = acute heart failure; ARDS = acute respiratory distress syndrome. Table S2: ICD-10 Procedure Codes. LHC = left heart catheterization; RHC = right heart catheterization; SWAN = swan-ganz catheterization; PCI = percutaneous coronary intervention; CABG = coronary artery bypass graft; VA ECMO = Veno-arterial extracorporeal membrane oxygenation; MCS = percutaneous temporary mechanical circulatory support; IABP = intra-aortic balloon pump; pVAD = percutaneous ventricular assist device, LVAD = left ventricular assist device. Figure S1. Patients included in the study cohort. Figure S2: Sub-etiologies of arrhythmic (Panel A) and valvular (Panel B) related cardiogenic shock by patient sex. Figure S3: Admission and maximum SCAI stage during cardiogenic shock hospitalization. SCAI = Society for Cardiovascular Angiography and Interventions. Figure S4: Multivariate logistic regression assessing differences in therapeutic interventions according to patient sex. CABG = coronary artery bypass graft, IABP = intra-aortic balloon pump, LVAD = left ventricular assist device, PCI = percutaneous coronary intervention, VA-ECMO = venoarterial extracorporeal membrane oxygenation. Figure S5: Mortality associated factors in women and men treated with non-acute myocardial infarction related cardiogenic shock. BSA = Body surface area, CABG = coronary artery bypass graft, CS = cardiogenic shock, DM = Diabetes mellitus, HTN = hypertension, IABP = intra-aortic balloon pump, LHC = left heart catheterization, LVAD = left ventricular assist device, PAC = pulmonary artery catheter, PCI = percutaneous coronary intervention, RRT = renal replacement therapy, VA-ECMO = venoarterial extracorporeal membrane oxygenation.

## Abbreviation

The following abbreviations are used in this manuscript.

CS	Cardiogenic shock
CSWG	Cardiogenic shock working group
SCAI	Society for Cardiovascular Angiography and Intervention
MCS	Mechanical circulatory support
AMI-CS	Acute myocardial infarction-related cardiogenic shock
nonAMI-CS	non-acute myocardial infarction-related cardiogenic shock
ICD	Internation classification of diseases
CKD	Chronic kidney disease
CAD	Coronary artery disease
VA-ECMO	Venoarterial extracorporeal membrane oxygenation
mAFP	Microaxial flow pump
LVAD	Left ventricular assist device
VIS	Vasoactive-inotropic score
BARC	Bleeding Academic Research Consortium
RRT	Renal replacement therapy
SHARC	Shock Academic Research Consortium
ACHF	Acute on chronic heart failure
VCS	Valvular cardiogenic shock
HF-CS	Heart failure-related cardiogenic shock
PAC	Pulmonary artery catheter
IABP	Intra-aortic balloon pump
LHC	Left heart catheterization
PCI	Percutaneous coronary intervention
ICU	Intensive care unit
CABG	Coronary artery bypass graft
BSA	Body surface area
eGFR	Glomerular filtration rate
SNF	Skilled nursing facility
