# Supplementary material for: Sex Differences in Non-Acute Myocardial Infarction Cardiogenic Shock: Insights from the Northwell-Shock Registry
Source: J Clin Med. 2025 Jun 16;14(12):4274. doi: 10.3390/jcm14124274 (PMC12194614; doi:10.3390/jcm14124274)
Supplement: Supplementary file 1 [file jcm-14-04274-s001.zip › jcm-3687925-supplementary.pdf]

# Supplemental Materials

**Table S1.** ICD-10 Admission & Principal Diagnosis Codes Included in Each Etiology Group. *ICD = international classification of disease; AMI = acute myocardial infarction; AHF = acute heart failure; ARDS = acute respiratory distress syndrome.*

| Etiology | ICD-10 Diagnosis Codes | |
|---|---|---|
| AMI | I20.0, I20.9, I21.01, I21.02, I21.09, I21.11, I21.19, I21.21, I21.29, I21.3, I21.4, I21.9, I21.A1, I21.A9, I22.2, I24.9, I25.10, I25.110, I25.118, I25.119, I25.5, I25.9, I20.8, I22.0, I22.8, I24.8, I25.810, I23.0, I23.2, I23.3, I23.8, I24.0, I25.42, R93.1, T82.855A, T82.857A, T82.867A, Z82.49, Z98.61, R07.89, R07.9 I50.9 | |
| AHF | Heart Failure | I50.9, I09.81, I11.0, I13.0, I13.2, I50.1, I50.20, I50.21, I50.22, I50.23, I50.30, I50.31, I50.33, I50.40, I50.41, I50.43, I50.810, I50.811, I50.813, I50.814, I50.82, I97.130, I97.131, J81.0, J81.1, I50.84, I31.9, I40.0, I40.9, I42.0, I42.2, I42.8, I42.9, I51.4, I51.7, I51.81, O90.3, O99.42, Z94.1, Z95.811, B57.2, I40.8, I42.6, I42.7, O99.43 |
| | Arrhythmia | I47.1, I47.2, I48.0, I48.1, I48.19, I48.20, I48.3, I48.91, I48.92, I49.01, I49.3, I49.5, I49.8, I49.9, R00.0, R00.1, R00.2, I48.21, I44.1, I45.5, I45.9, R94.31, I44.2, I44.30 |
| | Valvular disorders | I05.0, I05.1, I05.9, I06.0, I07.1, I08.0, I08.1, I08.3, I34.0, I34.1, I34.8, I34.9, I35.0, I35.1, I35.2, I35.9, Q23.0, Q23.1, I05.2, I33.0, I33.9, I34.2, I35.8, I36.1, I37.1, I38 T82.03XA, T82.09XA, T82.228A, T82.6XXA, T82.01XA, T82.221A, T82.310A |
| Other | Spesis/Infection | A09, A32.7, A40.0, A40.1, A40.3, A40.8, A40.9, A41.01, A41.02, A41.1, A41.3, A41.4, A41.50, A41.51, A41.52, A41.53, A41.59, A41.81, A41.89, A41.9, A48.1, A49.9, B02.7, B19.20, B34.9, J06.9, J12.1, J12.9, J15.8, J15.9, J18.1, J18.9, J20.9, J85.1, O98.52, R65.21, T80.211A, T80.212A, T81.42XA, T81.44XA, U07.1, B20, B33.8, B37.7, J10.01, J10.08, J10.1, J12.89, J15.211, J15.6, J20.8, T80.218A, T80.29XA, T81.41XA, T81.43XA, T81.4XXA, T83.511A, T83.512A, T83.518A, T83.592A |
| | Surgical | I71.00, I71.01, I71.02, I71.03, I71.1, I71.2, I71.3, I71.4, I71.6, I71.8, I71.9, I74.09, I74.11, I77.810, I97.190, I97.51, I97.621, I97.790, I97.88, I97.89, Q25.43, Q25.49, T82.330A |
| | Pulmonary Embolism | I23.6, I26.02, I26.09, I26.90, I26.92, I26.93, I26.94, I26.99, I27.82, I82.220, I82.403, I82.409, I82.432, I82.441, I82.4Z2, I82.622, I87.8 |
| | Cardiac Arrest | I46.8, I46.9, I97.120, I97.121, I97.711 |
| | ARDS | J80, J96.00, J96.01, J96.02, J96.20, J96.21, J96.22, J96.90, J96.91, J96.92, J98.9, R06.03, R09.2 |
| | Kidney Disease | I12.0, I12.9, I13.10, N17.0, N17.8, N17.9, N18.3, N18.6, N18.9, N19 |

| | | |
|---|---|---|
| | Other Cardiac Diagnosis | D15.1, I25.3, I27.9, I30.8, I30.9, I31.1, I31.2, I31.3, I31.4, I51.1, I51.3, I51.89, I51.9, I73.9, I74.3, I74.2, I74.5, I74.9, I77.9, I99.8, M32.12, O9A.12, Q21.0, Q21.1, Q21.3, Q23.4, R09.89, R94.30, R94.39, T82.110A, T82.118A, T82.120A, T82.190A, T82.198A, T82.41XA, T82.42XA, T82.518A, T82.524A, T82.594A, T82.598A, T82.818A, T82.838A, T82.897A, T82.898A, T82.9XXA, Z45.010, Z45.02, Z95.810 |
| | Other Non-Cardiac Diagnosis | 000.00, A04.72, A15.0, A24.9, A26.7, A48.0, B01.11, B19.9, B37.81, B96.5, C13.9, C15.4, C15.9, C16.8, C17.0, C18.0, C18.4, C18.6, C18.7, C18.9, C19, C21.8, C25.0, C25.2, C34.02, C34.11, C34.12, C34.90, C34.91, C34.92, C38.0, C40.21, C48.0, C49.3, C54.1, C55, C61, C64.1, C67.9, C70.1, C71.9, C77.1, C78.00, C78.01, C78.6, C78.7, C79.31, C79.51, C7A.1, C80.1, C82.90, C83.30, C83.32, C83.39, C84.48, C85.10, C85.11, C85.18, C85.89, C85.90, C85.91,C85.95, C86.5, Other C90.00, C91.00, C91.10,C91.50, C91.51, C91.92, C92.00, C92.Z0, C95.90, D32.0, D35.01, D35.1, D35.2, D3A.090, D3A.8, D46.9, D50.0, D57.00, D57.01, D59.1, D59.13, D61.810, D61.818, D62, D64.9, D65, D68.32, D68.9, D69.3, D69.6, D70.9, D72.829, D86.0, D86.89, E03.5, E05.01, E10.10, E11.00, E11.10, E11.22, E11.43, E11.51, E11.52, E11.621, E11.649, E11.65, E11.69, E15, E16.2, E22.2, E32.8, E66.01, E72.20, E80.6, E83.42, E83.51, E83.52, E83.59, E85.2, E85.4, E85.81, E85.82, E86.0, E86.9, E87.0, E87.1, E87.2, E87.5, E87.70, E87.79, E87.8, E88.09, E88.3, F10.10, F10.120, F10.221, F10.231, F10.239, G00.8, G04.81, G06.2, G40.101, G40.401, G40.89, G40.901, G40.909, G45.1, G45.9, G58.8, G70.01, G89.11, G92, G93.1, G93.40, G93.41, G93.49, G93.6, G93.89, G95.29, H50.07, H53.9, I10, I16.0, I16.1, I27.0, I27.20, I27.21, I27.24, I27.29, I28.8, I60.00, I60.11, I60.2, I60.31, I60.4, I60.7, I60.8, I60.9, I61.1, I61.5, I61.8, I61.9, I62.00, I62.01, I62.9, I63.12, I63.211, I63.212, I63.231, I63.233, I63.311, I63.40, I63.411, I63.433, I63.50, I63.511, I63.512, I63.519, I63.521, I63.59, I63.89, I63.9, I65.1, I65.22, I65.29, I67.1, I67.89, I69.398, I70.201, I70.203, I70.211, I70.222, I70.232, I70.234, I70.261, I70.421, I72.4, I72.9, I77.71, I77.74, I81, I85.01, I96, J36, J39.8, J43.9, J44.0, J44.1, J45.51, J45.901, J45.902, J68.1, J69.0, J69.8, J70.2, J84.10, J84.112, J85.2, J86.0, J86.9, J90, J91.8, J93.0, J93.9, J94.2, J95.03, J95.811, J95.830, J95.89, J98.4, J98.51, J98.59, J98.8, K11.20, K11.21, K20.91, K22.3, K25.1, K25.4, K25.5, K25.6, K26.0, K26.4, K26.5, K26.6, K29.01, K31.1, K31.811, K31.82, K35.80, K40.30, K41.40, K42.0, K43.0, K43.1, K43.2, K43.3, K43.5, K43.6, K43.9, K45.0, K45.8, K46.0, K52.9, K55.019, K55.039, K55.059, K55.1, K55.21, K55.8, K55.9, K56.1, K56.2, K56.41, K56.5, K56.50, K56.60, K56.601, K56.609, K56.69, K56.691, K56.7, K57.20, K57.31, K57.33, K57.40, K57.91, K57.92, K58.0, K59.00, K62.5, K62.89, K63.1, K65.1, K65.9, K66.1, K66.8, K70.30, K70.31, K70.40, K70.9, K71.10, K71.11, K72.00, K72.01, K72.90, K74.60, K74.69, K75.0, K76.1, K76.7, K80.00, K80.20, K80.30, K80.31, K80.50, K80.71, K81.0, K81.9, K82.8, K83.09, K83.3, K85.10, K85.12, K85.20, K85.21, K85.90, K86.1, K91.840, K92.0, K92.1, K92.2, K92.9, K95.89, L02.214, L02.511, L02.91, L03.115, L03.116, L03.119, L03.90, L08.9, L29.9, L76.22, L76.34, L89.150, L89.159, L97.101, L97.509, L97.519, L98.492, M00.011, M10.9, M17.11, M17.12, M25.511, M25.519, M25.551, M25.561, M31.1, M33.12, M35.81, M35.9, M40.205, |

| | | |
|---|---|---|
| | | M40.209, M43.9, M46.27, M48.06, M48.061, M48.062, M48.56XA, M50.20, M51.16, M54.16, M54.2, M54.9, M62.82, M66.862, M71.551, M72.6, M79.602, M79.604, M79.605, M79.606, M79.609, M79.662, M79.671, M79.89, M80.052A, M80.88XA, M84.40XA, M84.551A, M84.552A, M84.562A, M84.58XA, M86.10, M86.171, M86.642, M89.18, M97.11XA, N05.8, N10, N12, N13.6, N14.1, N20.0, N25.9, N28.9, N30.01, N30.90, N32.1, N32.89, N36.5, N39.0, N39.3, N48.89, N49.3, N76.89, N81.3, N82.3, N82.4, N92.0, N93.9, N95.0, O75.3, O9A.212, P76.9, Q27.33, Q43.0, R04.0, R04.2, R04.89, R05, R06.2, R06.89, R09.02, R10.11, R10.13, R10.31, R10.84, R10.9, R11.0, R11.10, R11.15, R11.2, R13.10, R13.13, R14.0, R17, R18.8, R19.00, R19.01, R19.7, R22.2, R22.43, R23.0, R25.1, R26.2, R26.81, R26.89, R26.9, R29.6, R29.810, R29.898, R29.90, R31.0, R31.9, R40.1, R40.20, R40.4, R41.0, R41.82, R41.89, R42, R47.01, R47.1, R47.81, R50.9, R51, R52, R53.1, R53.81, R53.83, R55, R56.1, R56.9, R57.1, R57.8, R57.9, R58, R59.1, R60.0, R60.1, R60.9, R62.7, R63.0, R63.8, R68.0, R68.89, R73.9, R74.0, R74.01, R74.8, R77.8, R78.81, R79.1, R79.89, R91.1, R91.8, R94.8, S00.83XA, S01.81XA, S02.2XXA, S02.80XA, S06.339A, S06.4X0A, S06.5X0A, S06.5X9A, S06.6X0A, S06.6X9A, S09.90XA, S12.111A, S12.120A, S15.121A, S21.431A, S22.009A, S22.069A, S22.081A, S22.20XA, S22.31XA, S22.39XA, S22.41XA, S22.42XA, S22.43XA, S22.49XA, S27.1XXA, S32.19XA, S32.511A, S32.591A, S32.599A, S32.810A, S36.031A, S36.032A, S36.899A, S39.91XA, S42.201K, S46.221A, S70.11XA, S72.001A, S72.002A, S72.009A, S72.012A, S72.021A, S72.041A, S72.091A, S72.092A, S72.111A, S72.141A, S72.142A, S72.145A, S72.21XA, S72.341A, S72.401A, S72.452A, S72.491A, S72.492A, S72.91XA, S73.005A, S76.112A, S80.11XA, S82.109C, S82.142C, S82.252A, S82.842C, S82.892B, T07, T14.8, T14.8XXA, T14.90, T14.90XA, T14.91XA, T17.820A, T17.890A, T17.908A, T17.920A, T17.928A, T22.391A, T24.311A, T30.0, T40.1X1A, T40.1X4A, T40.2X1A, T40.3X2A, T40.5X1A, T42.1X1A, T42.4X1A, T42.4X2A, T42.4X4A, T43.212A, T43.591A, T43.594A, T43.621A, T44.7X1A, T44.7X2A, T44.8X1A, T44.901A, T45.0X2A, T45.511A, T46.0X1A, T46.1X1A, T46.1X1S, T46.1X2A, T46.5X1A, T46.5X2A, T50.4X2A, T50.4X4A, T50.901A, T50.902A, T50.991A, T53.6X4A, T56.891A, T67.5XXA, T68.XXXA, T71.162A, T71.164A, T78.2XXA, T78.3XXA, T79.4XXA, T79.6XXA, T79.A0XA, T79.A21A, T79.A22D, T81.30XA, T81.31XA, T81.32XA, T81.595A, T81.718A, T81.719A, T81.83XA, T81.89XA, T81.9XXA, T84.125A, T85.611A, T85.868A, T86.19, T86.5, T87.43, T87.44, T87.54, T87.89, T88.6XXA, V87.7XXA, Z04.3, Z20.828, Z34.80, Z41.8, Z45.2, Z47.33, Z51.11, Z51.5, Z51.89, Z68.42, Z82.3, Z86.711, Z90.10, Z92.81 |

**Table S2.** ICD-10 Procedure Codes. *LHC = left heart catheterization; RHC = right heart catheterization; SWAN = swan-ganz catheterization; PCI = percutaneous coronary intervention; CABG = coronary artery bypass graft; VA ECMO = Veno-arterial extracorporeal membrane oxygenation; MCS = percutaneous temporary mechanical circulatory support; IABP = intra-aortic balloon pump; pVAD = percutaneous ventricular assist device, LVAD = left ventricular assist device*

| Procedure | ICD-10 Procedure Codes | |
|---|---|---|
| LHC | 4A023N7, 4A023N8, B2111ZZ, B211YZZ, B2161ZZ, B216YZZ, B2151ZZ, B215YZZ, B2181ZZ, B218YZZ, B2131ZZ, B213YZZ, B21F1ZZ, B2121ZZ, B2101ZZ, B210110 | |
| PAC | 4A023N6, 4A023N8, B2141ZZ, B31S1ZZ, B31U1ZZ, B214YZZ, B2161ZZ, B216YZZ, B2171ZZ, 5A12012, B31TYZZ, 02HR32Z, 02HQ33Z, 02HR33Z, 02HP32Z, 02HQ02Z, 02HQ32Z, 4A133B3, 5A1221Z | |
| PCI | 027337Z, 027037Z, 027036Z, 02703FZ, 027035Z, 02703EZ, 0270346, 0270356, 027034Z, 0270456, 02703DZ, 02703Z6, 02703ZZ, 027237Z, 027236Z, 0272356, 0272346, 027234Z, 02723ZZ, 027137Z, 027136Z, 027135Z, 02713EZ, 0271376, 0271366, 0271356, 027134Z, 02713DZ, 02714E6, 02713ZZ, 02C03ZZ, X2C2361, 02C13Z6, X2C1361, 02C13ZZ, 0271346, 0273346, 0270366, 027046Z, 02C00ZZ | |
| CABG | 021309W, 02130Z9, 0210093, 0210099, 02100AW, 02100KW, 02100JW, 02100J3, 02100Z3, 02100A9, 02100Z9, 02100Z8, 02100AC, 02100ZC, 0212093, 0212099, 021209W, 0211093, 0211099, 02110AW, 021109W, 021009W, 02110KW, 02110JW, 02100K8, 02110Z9, 02110Z8, 02110AC, 0213099, 02100A8, 021009C, 02120Z9, 02110A8, 02110Z3 | |
| Valve Procedure | 025G3ZZ, 027F3ZZ, 02BF0ZZ, 02BG0ZX, 02BG0ZZ, 02BJ0ZZ, 02CF0ZZ, 02CG0ZZ, 02CJ0ZZ, 02QF0ZZ, 02QG0ZZ, 02QJ0ZZ, 02RF07Z, 02RF08Z, 02RF0KZ, 02RF38Z, 02RF3JZ, 02RG08Z, 02RG0JZ, 02RG0KZ, 02RH0KZ, 02RJ08Z, 02UF08Z, 02UF0JZ, 02UF0KZ, 02UG07Z, 02UG08Z, 02UG0JZ, 02UG3JZ, 02UJ0JZ, 0DBC8ZX, X2RF032, 02RG3JH, 02RH08Z, 02RJ38Z, 02UJ3JZ, 02UJ4JZ, 02VG0ZZ, 02WG0JZ | |
| MCS | IABP | 5A02210, 5A02110 |
| | pVAD | 5A0221D, 02HA3RZ, 02HA4RZ, 02HA3RJ, 02HA0RZ, 5A0211D, 02HA3RS |
| | VA ECMO | 5A15223, 5A1522G, 5A1522F, 5A02216 |
| | Protek Duo | 02HA3QZ |
| | LVAD | 02HA0QZ |
| Heart Transplant | 02YA0Z0 | |

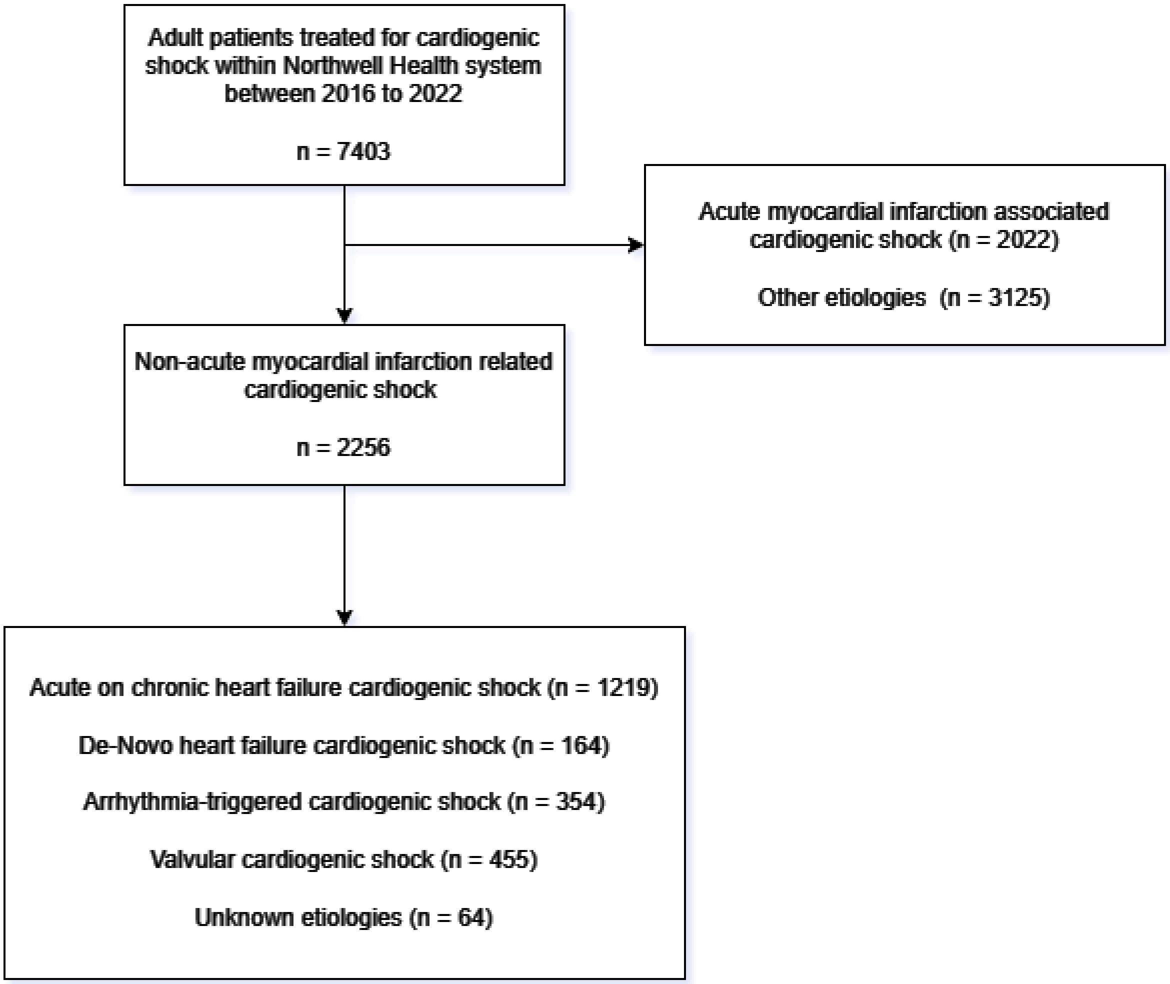


**Figure S1.** Patients included in the study cohort

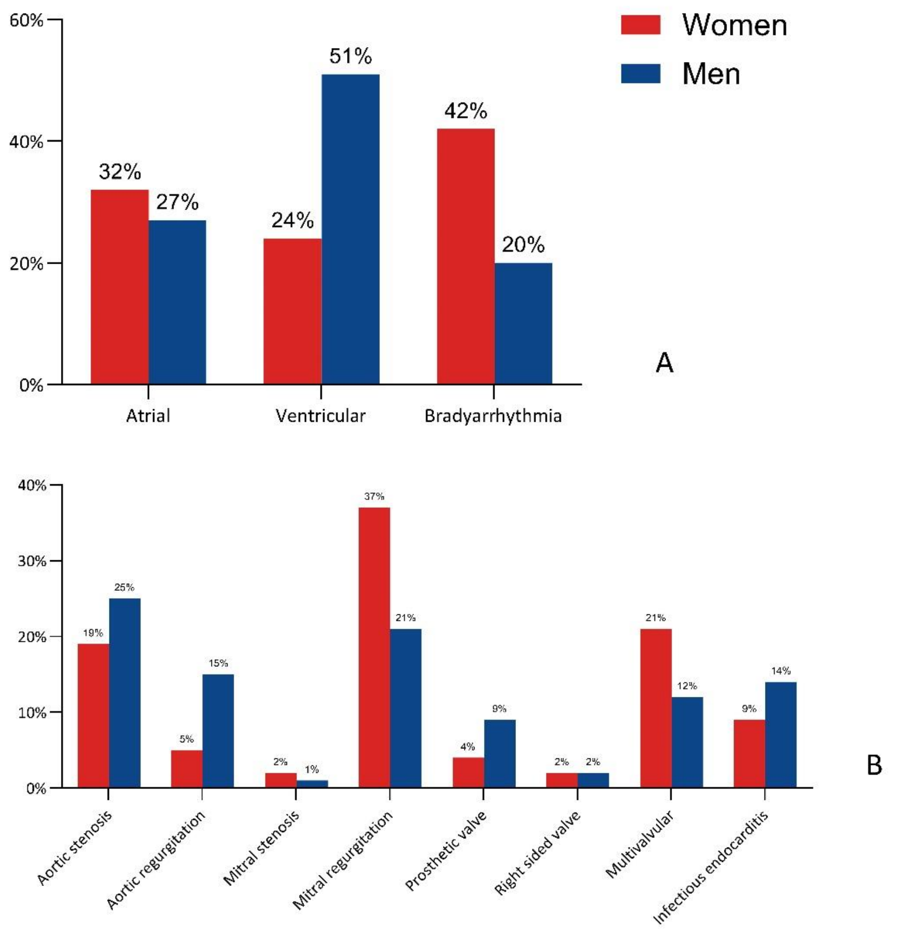


**Figure S2.** Sub-etiologies of arrhythmic (Panel A) and valvular (Panel B) related cardiogenic shock by patient sex

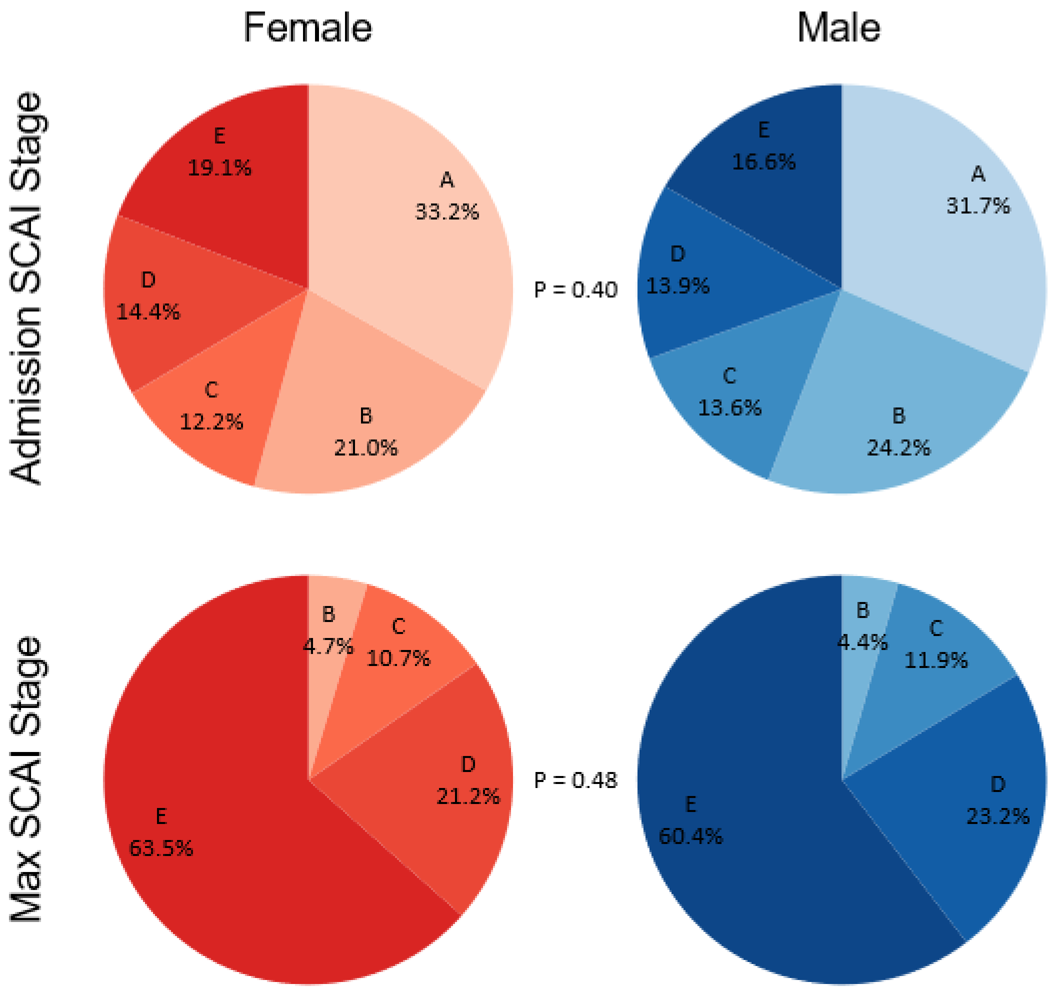


**Figure S3.** Admission and maximum SCAI stage during cardiogenic shock hospitalization. *SCAI = Society for Cardiovascular Angiography and Interventions*

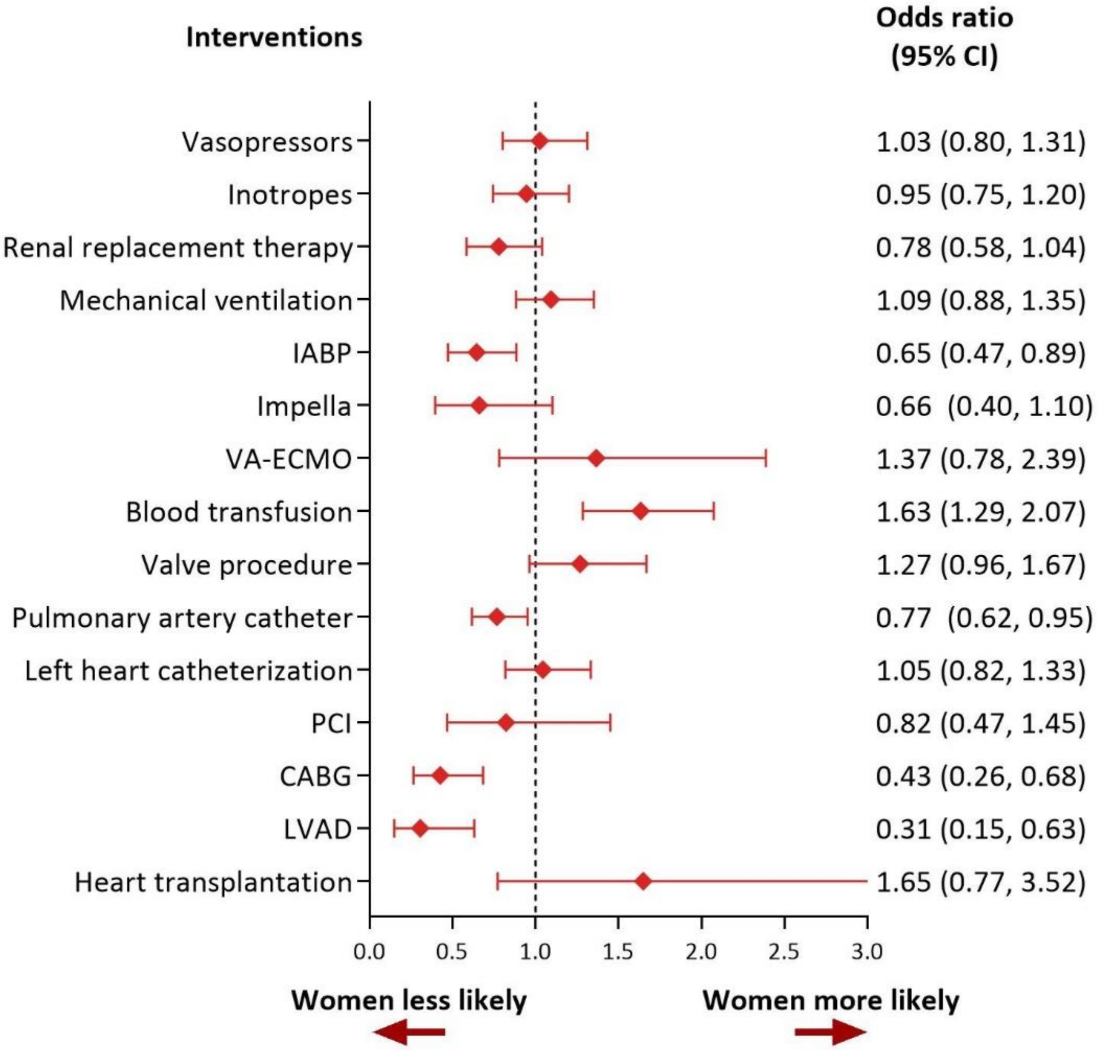


**Figure S4.** Multivariate logistic regression assessing differences in therapeutic interventions according to patient sex. *CABG = coronary artery bypass graft, IABP = intra-aortic balloon pump, LVAD = left ventricular assist device, PCI = percutaneous coronary intervention, VA-ECMO = venoarterial extracorporeal membrane oxygenation.*

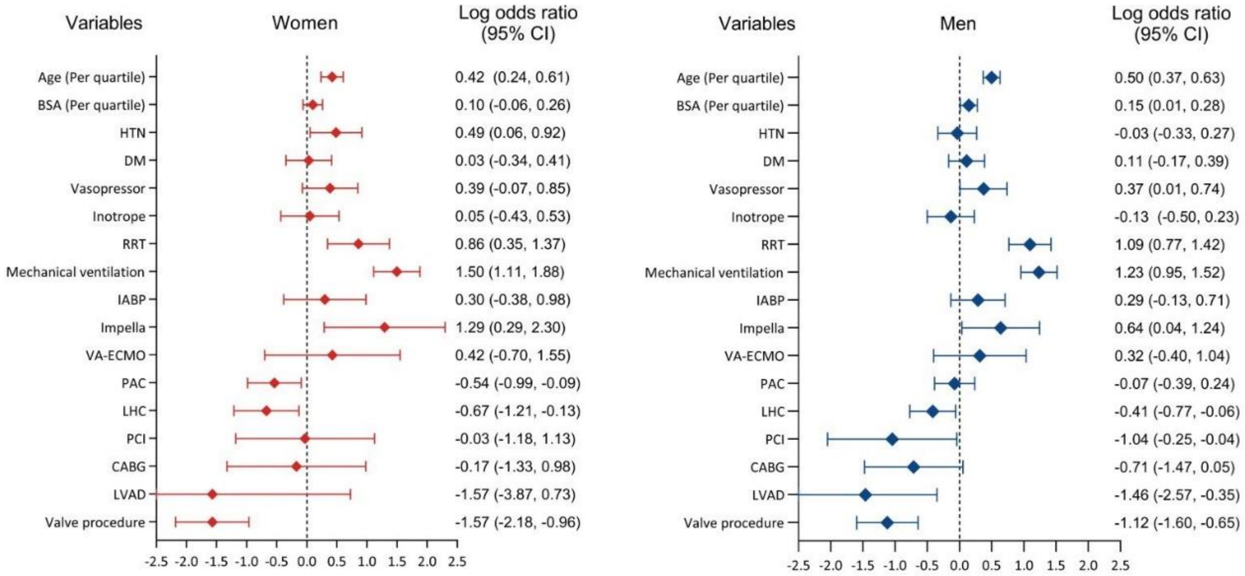


**Figure S5.** Mortality associated factors in women and men treated with non-acute myocardial infarction related cardiogenic shock. *BSA = Body surface area, CABG = coronary artery bypass graft, CS = cardiogenic shock, DM = Diabetes mellitus, HTN = hypertension, IABP = intra-aortic balloon pump, LHC = left heart catheterization, LVAD = left ventricular assist device, PAC = pulmonary artery catheter, PCI = percutaneous coronary intervention, RRT = renal replacement therapy, VA-ECMO = venoarterial extracorporeal membrane oxygenation*
